# Hypoxaemia as a Mortality Risk Factor in Acute Lower Respiratory Infections in Children in Low and Middle-Income Countries: Systematic Review and Meta-Analysis

**DOI:** 10.1371/journal.pone.0136166

**Published:** 2015-09-15

**Authors:** Marzia Lazzerini, Michela Sonego, Maria Chiara Pellegrin

**Affiliations:** 1 WHO Collaborating Centre for Maternal and Child Health, Institute for Maternal and Child Health IRCCS Burlo Garofolo, Via dell’Istria 65/1, 34137, Trieste, Italy; 2 University of Trieste, Piazzale Europa, 1 34127 Trieste, Italy; The Hospital for Sick Children, CANADA

## Abstract

**Objective:**

To evaluate the association between hypoxaemia and mortality from acute lower respiratory infections (ALRI) in children in low- and middle-income countries (LMIC).

**Design:**

Systematic review and meta-analysis.

**Study Selection:**

Observational studies reporting on the association between hypoxaemia and death from ALRI in children below five years in LMIC.

**Data Sources:**

Medline, Embase, Global Health Library, Lilacs, and Web of Science to February 2015.

**Risk of Bias Assessment:**

Quality In Prognosis Studies tool with minor adaptations to assess the risk of bias; funnel plots and Egger’s test to evaluate publication bias.

**Results:**

Out of 11,627 papers retrieved, 18 studies from 13 countries on 20,224 children met the inclusion criteria. Twelve (66.6%) studies had either low or moderate risk of bias. Hypoxaemia defined as oxygen saturation rate (SpO2) <90% associated with significantly increased odds of death from ALRI (OR 5.47, 95% CI 3.93 to 7.63) in 12 studies on 13,936 children. An Sp02 <92% associated with a similar increased risk of mortality (OR 3.66, 95% CI 1.42 to 9.47) in 3 studies on 673 children. Sensitivity analyses (excluding studies with high risk of bias and using adjusted OR) and subgroup analyses (by: altitude, definition of ALRI, country income, HIV prevalence) did not affect results. Only one study was performed on children living at high altitude.

**Conclusions:**

The results of this review support the routine evaluation of SpO2 for identifying children with ALRI at increased risk of death. Both a Sp02 value of 92% and 90% equally identify children at increased risk of mortality. More research is needed on children living at high altitude. Policy makers in LMIC should aim at improving the regular use of pulse oximetry and the availability of oxygen in order to decrease mortality from ALRI.

## Introduction

Acute lower respiratory infections (ALRI), such as pneumonia and bronchiolitis, are the leading cause of morbidity and mortality in children under five years of age. According to recent estimates, every year about 120–156 million cases of ALRI occur globally with approximately 1.4 million resulting in death. More than 95% of these deaths occur in low and middle income countries (LMIC)[1–3].

Currently there is a significant debate on how to improve the identification of cases of “severe ALRI“, and on which are the best prognostic factors that can be used in routine care to identify children with a higher risk of death [4–6].

Pulse oximetry is a non-invasive, simple and reliable method for measuring the saturation of arterial haemoglobin with oxygen, and it can detect desaturation under a variety of conditions [7–8].

According to a systematic review, hypoxaemia as detected with pulse oximetry has been observed in 13% of children with WHO-defined pneumonia requiring hospitalisation (severe and very severe classifications). This corresponds to at least 1.5 to 2.7 million annual cases of hypoxaemic pneumonia presenting to health-care facilities [9].

Although several research papers have suggested that hypoxia may be associated with increased odds of death in children with ALRI, no recent systematic review has synthetised the evidence in this regard. A previous systematic review dates back to year 2001, while a significant amount of literature has been published after that date [10]. The aim of this work was to systematically review and meta-analyse the evidence on the association between hypoxia, defined using different cut-offs, and ALRI mortality in children in LMIC.

A better understanding of this association may inform both policy makers, health staff directly involved with clinical management of ALRI in children, and researchers.

## Methods

### Search strategy and eligibility criteria

In conducting this review we followed the guidelines reported in the PRISMA (Preferred Reporting Items for systematic reviews and meta-analyses) [11]. and the MOOSE (Meta-analysis of Observational Studies) [12]. A protocol including detailed methods of the review was developed before starting the review.

We searched up to January 2015 the following databases: MEDLINE through Pubmed (from 1956); Embase through OVID (from 1974); Global Health Library (WHO website, no date restrictions), LILACS through the Virtual Health Library (no date restrictions); Science Citation Index Expanded (SCI-EXPANDED) through Web of Science (from 1992); Social Sciences Citation Index (SSCI) through Web of Science (from 1992). The search strategy is reported in **Table 1**. Manual searches of reference lists were also performed. We did not apply any language restrictions.

**Table 1:** Search strategy.

**MEDLINE (Pubmed)- vecchia**
((("pneumonia"[MeSH Terms] OR pneumonia[Text Word] OR "respiratory infection"[All Fields] OR "respiratory infections"[All Fields] OR "lower respiratory infection"[All Fields] OR "lower respiratory tract infections"[All Fields] OR "Bronchiolitis"[Mesh] OR "Bronchiolitis, Viral"[Mesh] OR bronchiolitis [All Fields]) AND ("child"[MeSH Terms] OR children[Text Word] OR "pediatrics"[MeSH Terms] OR "paediatrics"[All Fields] OR "paediatric"[All Fields] OR "Infant"[Mesh] OR infant [Text Word] OR newborn[Text Word] OR newborns [Text Word] OR neonate[Text Word] OR neonates [Text Word] OR infants [Text Word]) AND ("risk factors"[MeSH Terms] OR "risk factor"[Text Word] OR "risk factors"[All Fields] OR determinant[All Fields] OR determinant[All Fields] OR determinants[All Fields] OR predictor[All Fields] OR predictors[All Fields] OR "predictive value"[All Fields] OR "risk"[MeSH Terms] OR "risk"[All Fields]) AND ("mortality"[Subheading] OR "mortality"[MeSH Terms] OR mortality[Text Word] OR "death"[MeSH Terms] OR "death"[All Fields] OR fatality[All Fields] OR fatal[All Fields] OR ("death"[MeSH Terms] OR "death"[All Fields] OR "deaths"[All Fields]))) **OR** ((("pneumonia"[MeSH Terms] OR pneumonia[Text Word] OR "respiratory infection"[All Fields] OR "respiratory infections"[All Fields] OR "lower respiratory infection"[All Fields] OR "lower respiratory tract infections"[All Fields] OR "Bronchiolitis"[Mesh] OR "Bronchiolitis, Viral"[Mesh] OR bronchiolitis [All Fields]) AND ("child"[MeSH Terms] OR children[Text Word] OR "pediatrics"[MeSH Terms] OR "paediatrics"[All Fields] OR "paediatric"[All Fields] OR "Infant"[Mesh] OR infant [Text Word] OR newborn[Text Word] OR newborns [Text Word] OR neonate[Text Word] OR neonates [Text Word] OR infants [Text Word]) AND (hypoxemia OR hypoxaemia OR hypoxia OR “oxygen saturation”) AND ("mortality"[Subheading] OR "mortality"[MeSH Terms] OR mortality[Text Word] OR "death"[MeSH Terms] OR "death"[All Fields] OR fatality[All Fields] OR fatal[All Fields] OR ("death"[MeSH Terms] OR "death"[All Fields] OR "deaths"[All Fields])))
**GLOBAL HEALTH LIBRARY (WHO web site)**
((pneumonia OR "respiratory infection" OR "respiratory infections" OR Neumoni$ OR Infeccao Respiratoria OR Infeccion Respiratoria OR Infeccoes Respiratorias OR Infecciones Respiratorias) AND (child$ OR infant$ OR pediatric$ OR paediatric$ OR Nino$ OR Crianca$ OR infant$ OR Pediatric$ OR newborn$ OR neonat$ OR "recien nacido" OR "recem nacido") AND (hypoxemia OR hypoxaemia OR hypoxia OR “oxygen saturation” OR"risk factors" OR "risk factor" OR determinant$ OR predict$ OR risk$) AND (mortality OR death$ OR fatal$ OR letal$ OR outcome$ OR mortalidad OR mortalidade OR muerte OR Morte OR desenlace$ OR obito$))
**LILACS (Virtual Health Library)**
(pneumonia OR "respiratory infection" OR "respiratory infections" OR Neumoní$ OR Infecção Respiratoria OR Infección Respiratoria OR Infecçoes Respiratorias OR Infecciones Respiratorias OR bronchiolitis) (child$ OR infant$ OR pediatric$ OR paediatric$ OR Niño OR ninos OR Criança OR Crianças OR infantil OR infantiles OR Pediátrico OR Pediátricos) (mortality OR death$ OR fatal$ OR letal$ OR outcome$ OR mortalidad OR mortalidade OR muerte OR Morte OR desenlace$)
**SCI-EXPANDED and SSCI (Web of Science)**
((pneumonia OR “respiratory infection” OR “respiratory infections” OR "lower respiratory tract infections" OR "bronchiolitis") AND (child* OR pediatric* OR paediatric* OR infant* OR newborn* or neonat*) AND (“risk factors” OR “risk factor” OR predictor* OR risk* OR determinant* OR "predictive value") AND (mortality OR death* OR fatal*)) **OR** ((pneumonia OR “respiratory infection” OR “respiratory infections” OR "lower respiratory tract infections" OR "bronchiolitis") AND (child* OR pediatric* OR paediatric* OR infant* OR newborn* or neonat*) AND (“hypoxemia” OR hypoxaemia OR hypoxia OR “oxygen saturation”) AND (mortality OR death* OR fatal*))
**EMBASE**
1)pneumonia/co, dm, dt, ep, et, pc, th [Complication, Disease Management, Drug Therapy, Epidemiology, Etiology, Prevention, Therapy];2) (children or infant*OR childhood or preschool*).mp. [mp = title, abstract, subject headings, heading word, drug trade name, original title, device manufacturer, drug manufacturer, device trade name, keyword]; 3) limit 2 to human; 4) 1 and 3; 5) developing countries.mp. or developing country/; 6) (Asia* or Africa* or South America).mp. [mp = title, abstract, subject headings, heading word, drug trade name, original title, device manufacturer, drug manufacturer, device trade name, keyword]; 7) LMIC.mp.; 8) "low and middle income countries".mp.; 9) 5 or 6 or 7 or 8; 10) 4 and 9.

Observational studies were eligible for inclusion if they reported the association between death from ALRI and hypoxaemia, in children under 5 years of age in LMIC, as defined by the World Bank [13]. Both studies at hospital level and in the community were included. Studies reporting selectively on children with very specific co-morbidities, such as studies on children with cancer, organ transplant, burns, ventilator-acquired pneumonia, SARS or avian flu were excluded. Studies with less than five events (deaths) were also excluded.

### Data collection

Studies were selected for inclusion by two independent authors (ML and MS). Any disagreement was resolved through discussion. The full text of all eligible citations was examined in detail. Seven authors were contacted for additional information and four provided additional data, and/or clarifications on the published data.

Two authors (MS and MCP) extracted data from included studies, using a pre-piloted data-extraction form. Disagreements were resolved by discussion between the two authors and consensus with a third author (ML). We extracted information regarding: country, setting and altitude where the study was performed (altitude, if not specified in the study was attributed based on the altitude of the location were the study was performed); period of the study; definition of hypoxaemia; definition for ALRI; characteristics of the population; study design; sample size; type of analysis performed (univariate or multivariate); confounders.

We extracted both unadjusted and adjusted OR or risk ratios (RR), and crude numbers. When possible, we converted RR to OR, using a formula for computing OR from RR [14]. To avoid mistakes due to data manipulation, data were first extracted as in the original paper, and then converted as needed.

In studies also including children over five years of age, only data on children under five were extracted. If sorting was not possible, we only included the study if at least 80% of the children were under five years old.

Single studies that reported on cut-offs of oxygen saturation rate (SpO2) that could not be grouped with other studies were grouped as more appropriate to improve the informativeness of results (**Table 2**, notes).

**Table 2:** Characteristics of included studies.

Author and year of publication	Type of study	Country	Period of study	Setting (source population)	Altitude (meters above sea level)	Cut off of Sp02 used	Diagnostic criteria for ALRI	Age group (months)	Other characteristics of the study population	ALRI mortality+	N° of children evaluated for hypoxemia
Chisti 2011	CO	BANGLADESH	2007	Hospital (Urban)	4	90%	Clinical WHO	0–59	All severe ALRI; All ALRI and diarrhoea	12.1	198
Chisti 2013	CC	BANGLADESH	2011–2012	PICU (Urban)	4	90%	Radiological	0–59	All severe ALRI; All severe acute malnutrition	─	140
Demers 2000	CO	CENTRAL AFRICAN REPUBLIC	1996–1997	Hospital (Urban)	386	Various*	Clinical WHO	0–59	-	12.5	392
Djelantik 2003	CO	INDONESIA	1999–2001	Three Hospitals (Rural)	26	85%	Clinical WHO	0–23	All severe ALRI	11.6	4306
Duke 2001	CO	PNG	1998–1999	Hospital (Rural)	1600	Various*	Clinical WHO combined with PNG guidelines	1–59	All severe ALRI; All with Sp02<85%	6.5	703
Graham 2011	CO	MALAWI	2005–2006	Hospital (Urban)	1039	90%	Clinical WHO	2–156 (12%>60)	All severe ALRI; HIV infected >50%	10.1	327
Junge 2006	CO	GAMBIA	1993–1994	Hospital (Urban)	29	Various*	Clinical	41%<12	-	4.6	436
Mc Nally 2007	CO	SOUTH AFRICA	2001–2002	Hospital (Urban)	22	92%	Clinical WHO	1–59	All severe ALRI; >50% HIV infected	15.1	358
Mwaniki 2009	CO	KENYA	2002–2005	Hospital (Rural)	Sea level	90%	Clinical WHO	0–59	-	7.8	5489
Nantanda 2008	CO	UGANDA	2005–2006	Hospital (Urban)	1260	92%	Clinical WHO	2–59	All severe ALRI	15.3	157
Nantanda 2014	CO	UGANDA	2011–2012	Hospital (Urban and Rural)	1260	92%	Clinical WHO °	2–59	Severe ALRI	3.6	614
Onyango 1993	CO	KENYA	1989	Hospital (Urban)	1670	90%	Clinical	0–36	-	10.0	209
Reed 2012	CO	SOUTH AFRICA	1998–2001	Hospital (Urban)	1753	90%	Clinical	<24	-	7.2%	4148
Rodríguez 2010	CC	COLOMBIA	2007–2008	Four Hospitals (Ward+PICU) (Urban)	2640	90%	ICD-10 death certificate for ALRI and clinical records	<60	-	─	226
Sigauque 2009	CO	MOZAMBIQUE	2004–2006	Hospital (Rural)	50	90%	Clinical WHO	0–23	All severe ALRI	6.5	584
Smyth 1998	CO	ZAMBIA	1994–1995	Hospital (Rural)	1150	90%	Clinical WHO	1–59	All severe ALRI	14.6	158
Usen 1999	CO	GAMBIA	1993–1995	Two Hospitals (Urban)	2	90%	Clinical	2–33	-	3.4	1072
Zhang 2013	CO	CHINA	2007–2010	PICU (Urban)	4	92%	Clinical WHO	0–60	All severe ALRI	5.8	707

* Demers used the following cut-offs: > = 95; 90–94.9; 85–89.9; <85. We grouped results as follows: > = 90; <90.

Duke used the following cut-offs: 70–84; 50–69; <50. We grouped results as follows: 70–84 vs < 70.

Junge used the following cut-offs: >94; 90–94; <90. We grouped results as follows: > = 90 vs <90.

° After admittance, children were further diagnosed using further South African Guideline for bronchiolitis, and GINA guidelines for asthma

### Assessment of risk of bias

Two review authors (MS and MCP) independently assessed the risk of bias using the Quality In Prognosis Studies (QUIPS) [15] tool 15 with minor adaptations **(Table A in S1 File**). The tool includes 32 key items divided into 6 domains: 1) Study participation; 2) Study attrition; 3) Prognostic factor measurement; 4) Outcome measurement; 5) Study Confounding; 6) Statistical Analysis and reporting. For each study, each individual key item was assessed, and each domain was graded in one of the following categories of risk of bias, based on whether the domain fully complied, partly complied, did not comply at all, or did not report in respect of the characteristic expressed by the items: 1) Low risk of bias; 2) Moderate risk of bias; 3) High risk of bias; 4) Unknown. The overall risk of bias for each single study was rated as follows **(Table B in S1 File)**: 1) Low—if all the six domains were scored as low, or if not more than two moderate or unknown risks of bias were identified with no high risk of bias; 2) Moderate—if moderate or unknown risk of bias was identified in three domains without any high risk of bias, or if a high risk of bias was identified in association with not more than one moderate or unknown risk of bias; 3) High—if a high risk of bias was identified in two or more domains independently from the grading in the other domains, or if moderate or unknown risk of bias was identified in four or more domains independently from grading in the other domains.

### Statistical analyses

When meta-analysis was possible and appropriate we generated a pooled OR using the inverse-variance weighting method. As we expected high heterogeneity among studies (in the population, in the definition of ALRI, in the definition of hypoxaemia, and the overall methodology), we selected *a priori* the DerSimonian and Laird random effect model [16], which accounts for intra- and inter- study variability.

Pooled data were presented in a forest plot, where studies are ordered chronologically; data from one study that could not be meta-analyzed were described in text.

We tested the null hypothesis that all studies evaluate the same true effect by the Cochran’s Q test, with p<0.05 considered statistically significant. The degree of heterogeneity between studies was assessed by visual inspection of the forest plots and I-squared (I^2^) statistic with its 95% confidence intervals. Heterogeneity was considered low for I^2^ values between 25%-50%, moderate for 50%-75%, and high for ≥75% [17].

### Sensitivity analyses and exploration of heterogeneity

We performed sensitivity analyses to examine the effect of removing the studies with high risk of bias and of using adjusted OR rather than the crude OR, when the former was available.

Using subgroup and metaregression analysis, we planned to explore the effect of the following study-level factors: a) Altitude (studies in populations living at 0–1,499 meters above sea level vs 1,500–2,499 meters vs > 2,500 meters); b) different definitions of ALRI used (WHO definition versus other definitions); c) income (low income countries versus lower middle income countries, as defined by the World Bank^13^ at the moment of the study start—no study was available for the category of upper middle income countries); d) HIV country prevalence (high HIV prevalence versus non-high HIV prevalence, where a high HIV prevalence was defined as a prevalence of HIV in the population of 15–49 year-olds at the time of the study > 5%.

We assessed potential publication bias and small study effects with funnel plots and the Egger test [18].

All statistical analysis was performed using Stata version 12 [19].

## Results

### Characteristics of the studies

**Fig 1:**
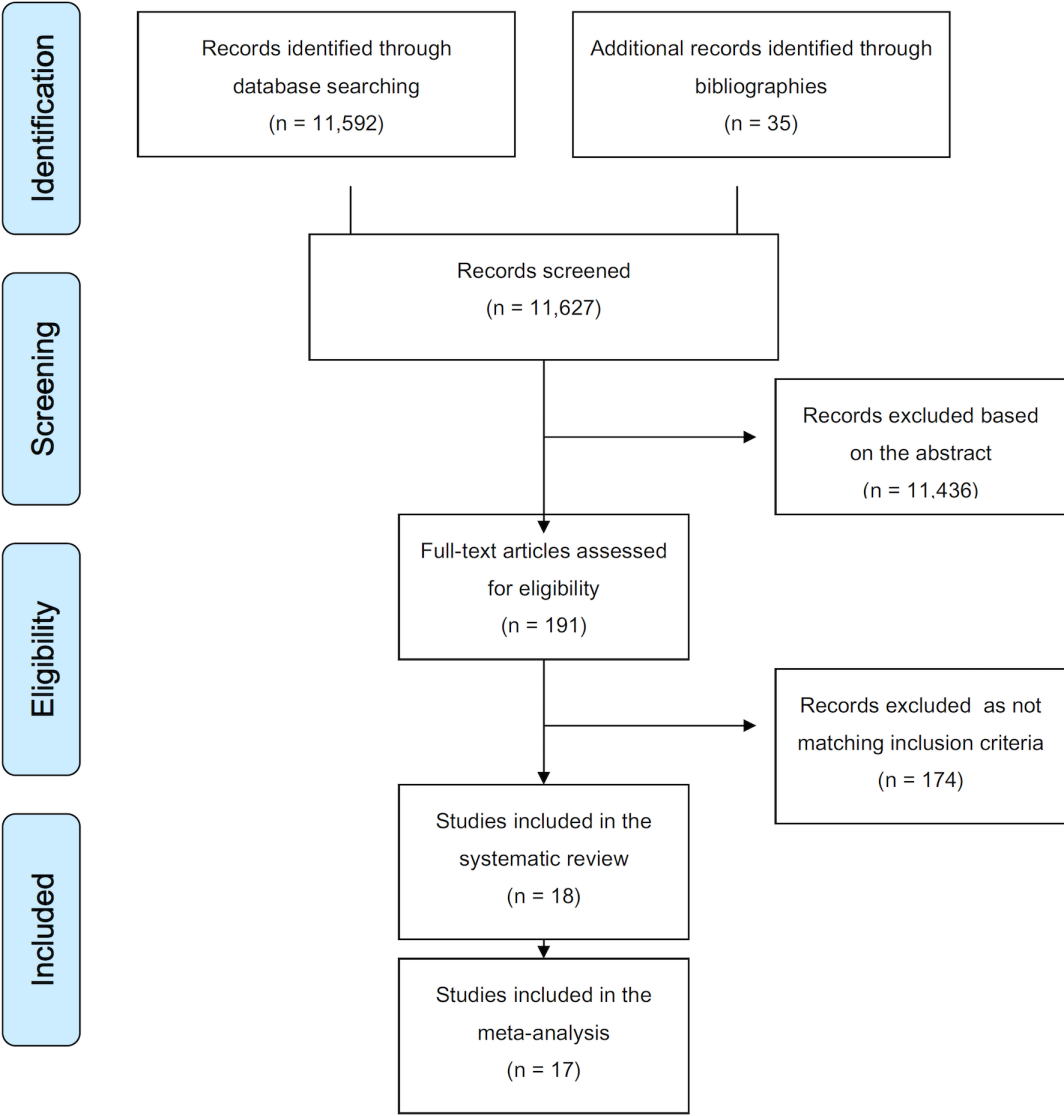
PRISMA 2009 Flow Diagram.

The systematic search yielded overall 11,627 records (**Fig 1**). Overall 18 studies [20–37] from 13 countries on 20,224 children met the inclusion criteria. Characteristics of the studies are summarized in **Table 2**.

All except three studies were published during the last 15 years. Twelve studies were performed in Africa (two studies in Gambia, Kenya, Uganda and South Africa; one each in Central African Republic, Malawi, Mozambique, Zambia), three in South East Asia (two in Bangladesh, one in Indonesia), two in Western Pacific Region (one in Papua New Guinea, one in China) and one in South America (Colombia). All studies were hospital-based, 12 were conducted in an urban setting setting, five in a rural setting, and one in both. Three studies were multi-centered. Fourteen studies were performed at an altitude of between 0–1,499 meters above sea level, three studies at an altitude between 1,500–2,499 meters, and one study at an altitude of > 2,500 meters above sea level (this study used as a cut-off of Sp02 a value of 90%).

Twelve studies contrubuted on the evaluation of hypoxaemia defined as a cut-off oxygen of SpO2 of 90%, four studies on a cut-off of 92%; one study on a cut-off of 85%, and one study on cut-offs of 70% versus 70–84%.

Overall, 12 studies used the WHO definition of pneumonia, while the other used other clinical criteria (five studies), radiological classification (one study) and ICD10 classification (one study). Eleven studies also included newborns. Eleven studies enrolled children with either severe or very severe pneumonia. Two studies in Bangladesh enrolled children who all presented either diarrhea (one study) or malnutrition (the other study) as a co-morbidity. In two studies in Malawi and South Africa respectively over half of enrolled children were HIV infected. Mortality rate in the included studies ranged from 3.4% to 15.3%.

One study [32] reported data separatly on HIV-positive and HIV-negative children, and was included in the meta-analysis as two sub-studies.

**Fig 2:**
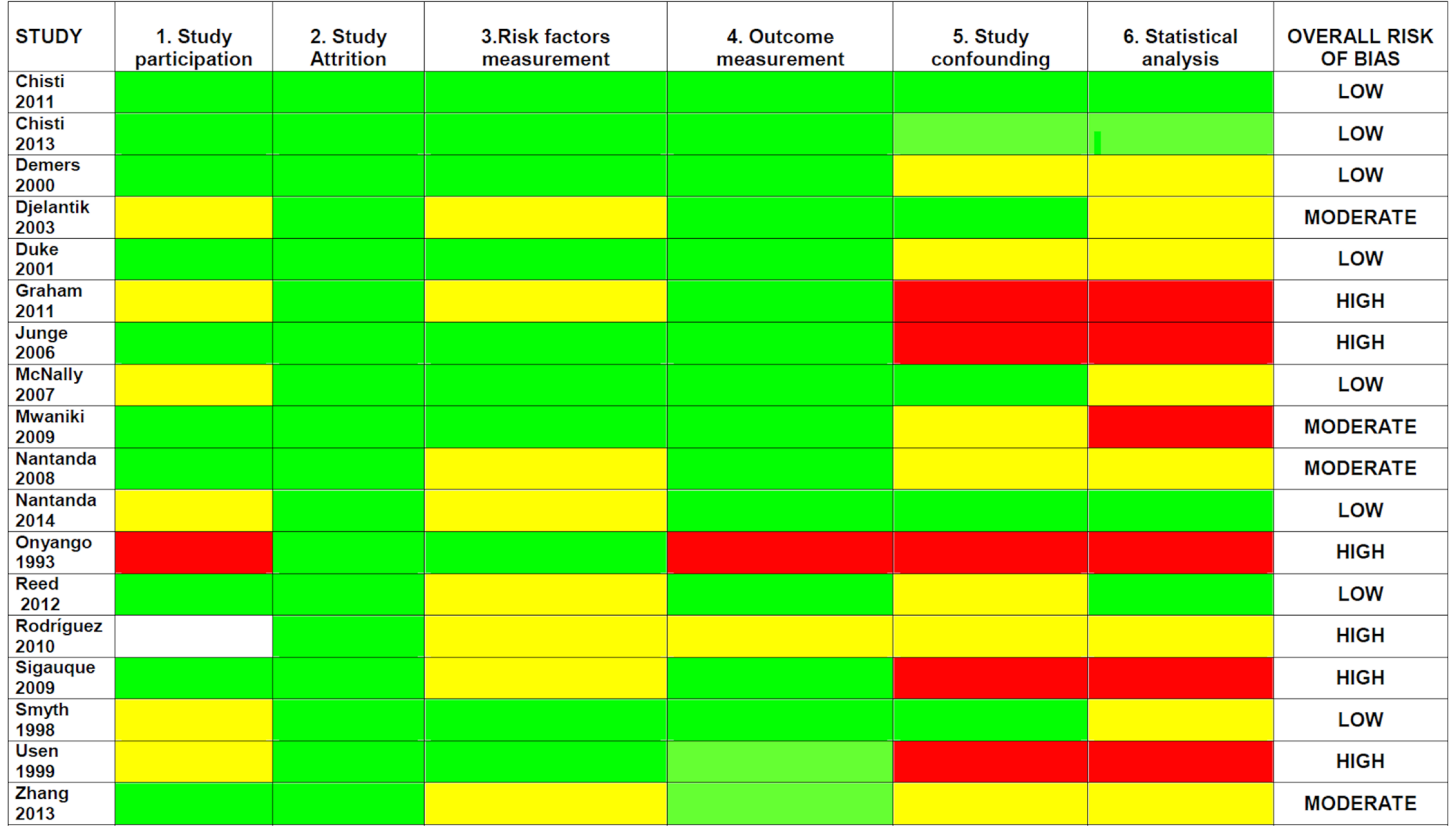
Risk of bias in the included studies. Red = High risk of bias. Yellow = Moderate risk of bias. Green = Low risk of bias. White = Unknown risk of bias.

Risk of bias for included studies is reported in **Fig 2**. Six studies were considered at high risk of bias, with lack of adjustment for confounding and other aspects of the statistical analysis being the most frequent risk of bias. The other 12 studies were considered either at low (eight studies) or at moderate (four studies) risk of bias.

### Results of the meta-analysis

**Fig 3:**
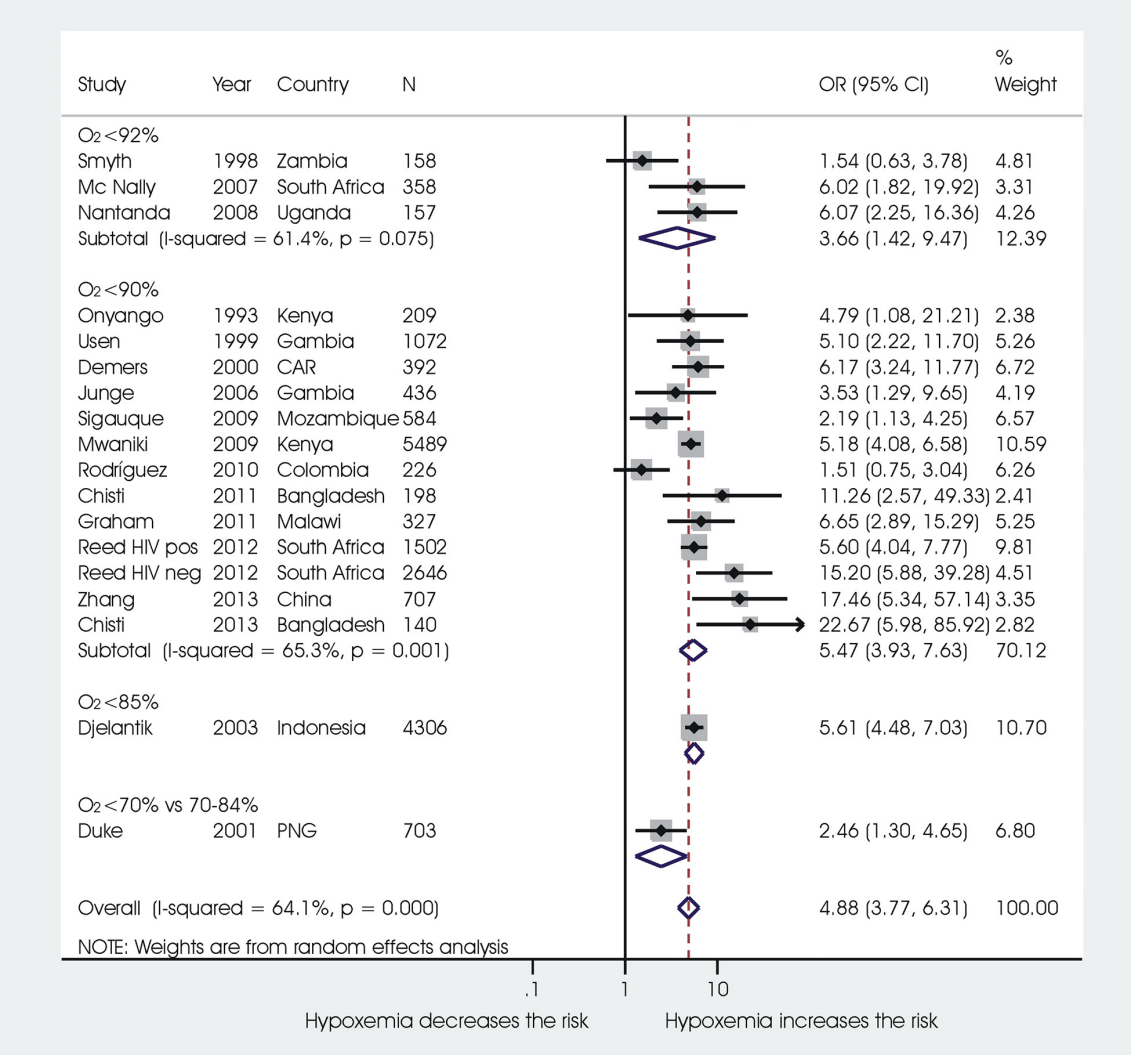
Association between hypoxemia and death. Notes: seventeen studies were able to be pooled in the meta-analysis. The remaining study that could not be meta-analysed used an SpO2 cut-off of 92% and found an HR of 12.2 (95% CI 1.6–92.0).29.

Results of the meta-analysis are reported in **Fig 3**. Seventeen studies were included in the meta-analysis.

Hypoxaemia defined with a cut-off for oxygen saturation rate (SpO2) below 90% was associated with significant increased odds of death from ALRI (OR 5.47, 95% CI 3.93 to 7.63) in 13 studies on 13,928 children.

An Sp02 below 92% compared to over 92% was associated with an increased risk of mortality of 3.66 (95% CI 1.42 to 9.47) in 3 studies on 673 children. Using the same cut-off of 92%, one study on 614 children found an HR of 12.2 (1.6–92.0) [29].

One study on 4,306 Indonesian children using a cut-off of SpO2 of 85% identified an OR of 5.61(95% CI 4.48 to 7.03).

A study in Papua New Guinea on 6,703 children comparing an SpO2 of < 70% to values 70–84% resulted in an OR of 2.46 (95% CI 1.30 to 4.65).

Heterogeneity of results was moderate in both the group of studies using a SpO2 cut-off value of 92% (3 studies, I2 = 61.4%), and in the group of studies using a cut-off value of 90% (13 studies, 65.3%).

### Sensitivity analyses

Results of sensitivity analyses are reported in **Table 3**. When only studies with either low or moderate risk of bias were included in the analysis (11 studies), hypoxaemia was overall still significantly associated with increased odds of mortalty (OR 5.71, 95% CI 4.34 to 7.50).

**Table 3:** Sensitivity analyses.

***Sensitivity analysis including only studies with low or moderate risk of bias***		
**N°. of studies with low or moderate risk of bias**	**N°. of studies included in the sensitivity analysis**	**Pooled OR (95%CI) when including in the meta-analysis only studies with low or moderate risk of bias**
12	11 (12 comparisons)	5.71 (4.34–7.50)
***Sensitivity analysis including only studies providing an adjusted OR***		
**N° of studies with adjusted OR available**	**N°. of studies included in the sensitivity analysis**	**Pooled OR (95%CI) of the 5 studies with adjusted OR**
4	4 (5 comparisons)	4.69 (2.55–8.61)
***Sensitivity analysis on all studies after substituting crude OR with adjusted OR for studies were adjusted OR was available***		
**N° of studies with adjusted OR available**	**N°. of studies included in the sensitivity analysis**	**Pooled OR (95%CI) after substituting crude OR with adjusted OR**
4	17 (18 comparisons)	4.66 (3.50–6.19)

Both when all studies were retained in the meta-analysis, but adjusted OR were used for studies providing them, and when only studies prividing an adjusted ORs were analysed (four studies), hypoxaemia was still significantly associated with increased odds of mortalty (OR = 4.66, 95% CI 3.50 to 6.19; OR = 4.69, 95% CI 2.55 to 8.61).

### Subgroup analyses and metaregression

**Fig 4:**
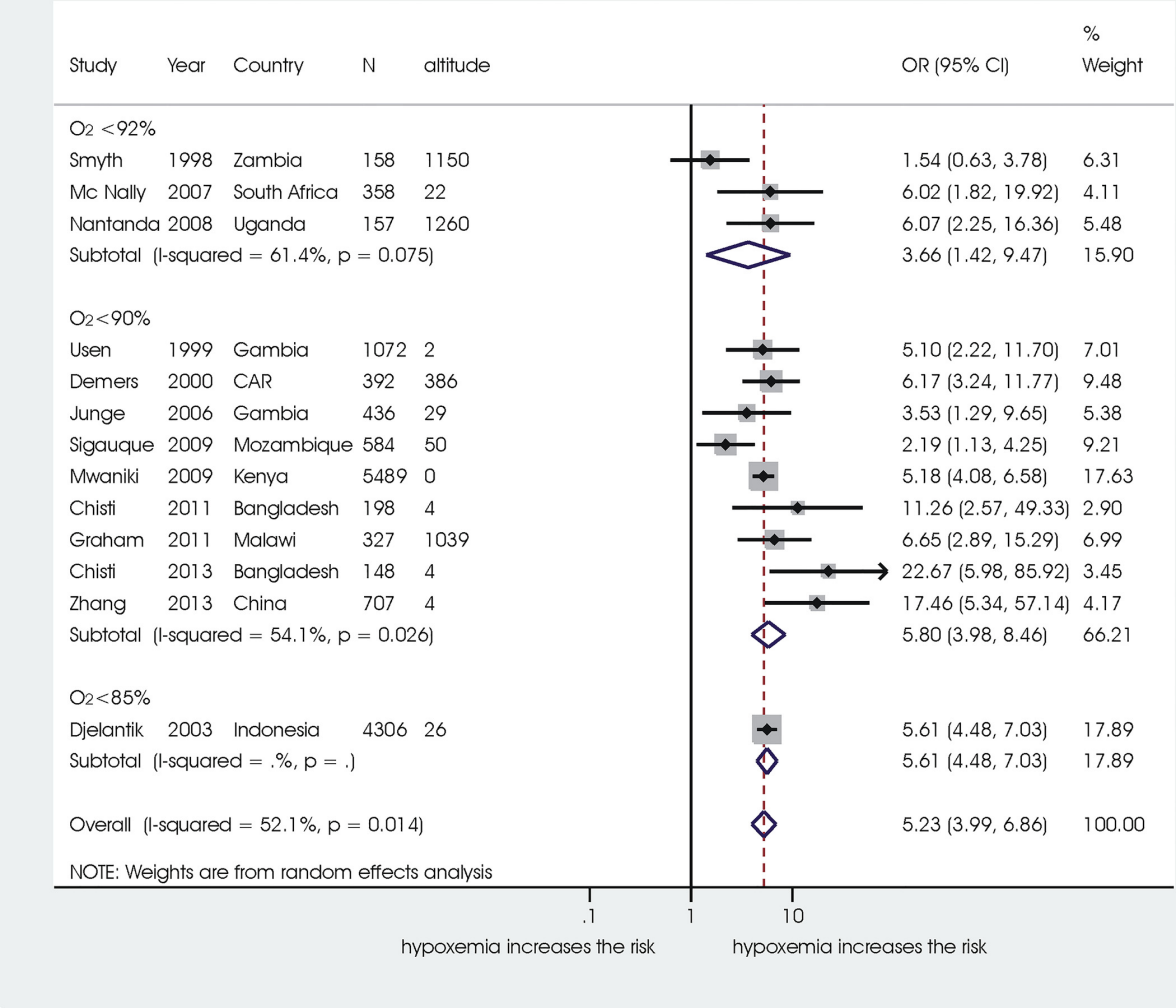
Subgroup analysis in the population living at 0–1,499 meters above sea level.

**Fig 5:**
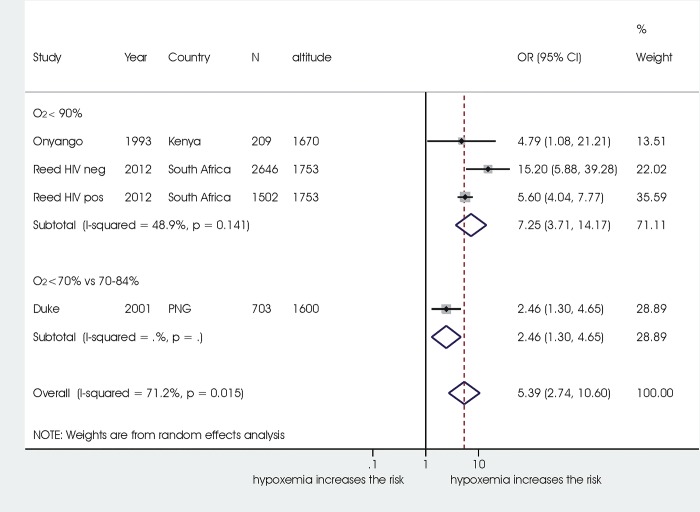
Subgroup analysis in the studies with a population living at 1,500–2,500 meters above sea level.

Results of the subgroup analysis and metaregression are reported in **Table 4.** Overall, subgroup analysis did not affect results. When studies were sub-grouped based on different altitudes where the studies were performed, the overall association between hypoxaemia and mortality persisted both in the subgroup studies in populations living at 0–1,499 meters above sea level (OR 5.23, 95%CI 3.99 to 6.86, 13 studies) and at 1,500–2,499 meters above sea level (OR 5.34, 95%CI 2.74 to 10.60, 3 studies, 4 comparisons). **Fig 4**and **Fig 5**report the subgroup analysis in detail: overall in both subgroups all explored SpO2 cut-offs were significantly associated with increased odds of mortality, without significant differences between different cut-offs, although in the subgroup of studies at 0–1,499 meters (**Fig 5**) there was a trend for higher risk of mortality with decreasing Sp02 cut-offs (92%: OR 3.66, 95% CI 1.42 to 9.47; 90%: OR 5.80, 95% CI 3.98 to 8.46). One only small study (255 children) reporting on a population in Colombia living at an altitude > 2,500 meters, and evaluating an Sp02 of 90%, did not identify a significant association with increased mortalty risk (OR 1.51, 95%CI 0.75 to 3.04).

**Table 4:** Subgroup analysis and metaregression.

Variable	Comparisons	N° of studies	Pooled OR	I2 (%)	p from meta-regression
Altitude	0–1499	13	5.23 (3.99–6.86)	52.1	
	1500–2499	3	5.39 (2.74–10.60)	71.2	
	>2500	1	1.51 (0.75–3.04)	─	
Diagnosis	WHO	10	5.06 (3.79–6.76)	54.6	
	Other diagnosis	7	4.84 (2.82–8.30)	73.8	0.811
Income	Low income countries	12	4.92 (3.78–6.40)	47.7	
	Lower-medium income countries	5	5.19 (2.64–10.21)	80.7	0.982
HIV prevalence	Low HIV prevalence	7	4.43 (2.57–7.64)	74.4	
	High HIV prevalence	9	4.33 (3.02–6.02)	47.8	0.971

When studies where subgrouped based on different diagnostic criteria for ALRI (WHO criteria vs other criteria), hypoxaemia was significanty associated with mortality in both subgroups (OR 5.06, 95% CI 3.79 to 6.76; OR 4.84, 95% CI 2.82 to 8.30, p = 0.811).

When studies where subgrouped based on incomes in the country (low vs lower-middle medium) hypoxaemia was significanty associated with mortality in both subgroups (OR 4.92, 95% CI 3.78 to 6.40; OR 5.19, 95% CI 2.64 to 10.21, p = 0.982)

When studies where subgrouped based on HIV prevalence in the country, hypoxaemia was significanty associated with mortality in both subgroups (OR 4.43, 95% CI 2.57 to 7.64; OR 4.33, 95% CI 3.02 to 6.02, p = 0.971).

### Publication bias

**Fig 6:**
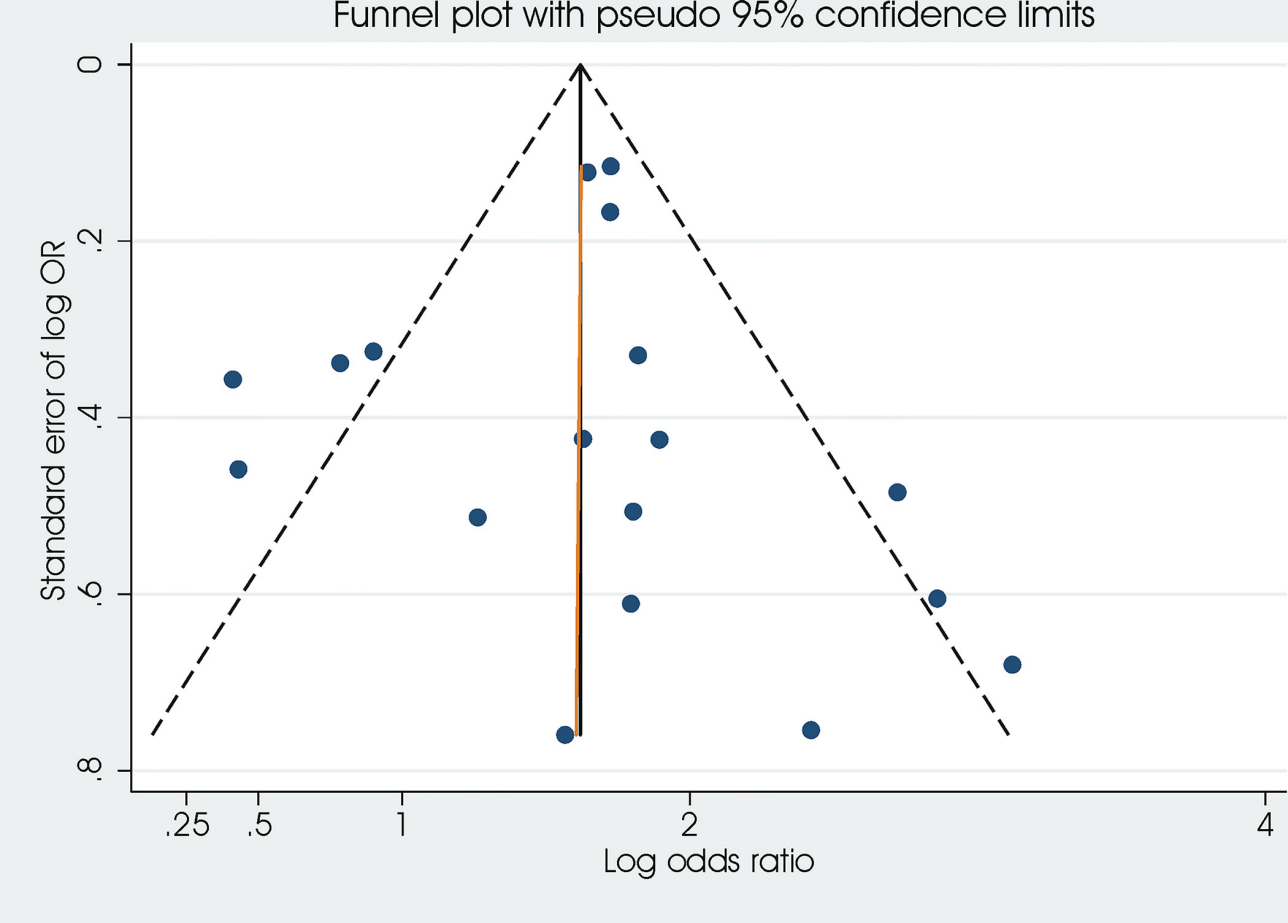
Funnel plot to explore publication bias.

The funnel plot is reported as **Fig 6**. Egger tests resulted in a p value of 0.971 (not suggestive of publication bias).

## Discussion

### Interpretation of the results

This systematic review synthesises the available evidence on the association between hypoxaemia and ALRI mortality in children in LMIC. Results from this review confirm the importance of measuring oxygen saturation in children with ALRI in order to identify children with higher risk of mortality. Overall, the association with hypoxaemia is well documented: the pooled analysis of 12 studies showed that children with SpO2 below 90% have a 5.4 fold increase in the risk of death, while 3 studies showed that children with SpO2 below 92% have a 3.6 fold increase in odds of death. Sensitivity analyses (including only studies with either low or moderate risk of bias and using adjusted OR) and subgroup analyses (by altitude, definition of ALRI, country income, HIV prevalence) did not change the results. Tests for publication bias did not suggest their existence.

A previous review published several years ago [10] identified only three studies reporting on hypoxaemia and mortality in children with ALRI. This review updates the current evidence-synthesis on this subject.

### Strengths and limitations

In performing this review we used a comprehensive search strategy without language restrictions; quality of retrieved studies was overall fair, without suggestion of publications bias. We were aware of the possibility of introducing bias at every stage of the review process and tried to minimise this aspect by following strict methods and standards suggested for systematic reviews [11,12,15].

Moderate heterogeneity of results among studies may be explained by heterogeneity in the characteristics of the studies, such as design, setting, type of population included, definition of ALRI, and power of the study (which may explain large confidence intervals in some instances). Subgroup analyses reduced part of the heterogeneity in some sub-groups, however heterogeneity may be also explained by other confounding factors affecting mortality rates, such as the different quality of care provided in the studies, and different algorithms used to treat hypoxaemia.

This review identifies a lack of data on the prognostic value of Sp02 in populations living at very high altitudes (> 2,500 meters above sea level).

Other limitations in this review are related to limitations in the designs of the included studies and in the availability of individual patient data: few studies in the analysis provided had adjusted OR after correction for other related risk factors, and no individual patient database was availble to generate pooled-adjusted OR. However, sensitivity analysis using adjusted ORs confirmed the primary results.

### Implication for policies and research

The results of this review support the routine evaluation of oxygen saturation rate for identifying children with ALRI at higher risk of death. This is in line with current WHO guidelines, which recommend determining the presence of hypoxaemia with pulse-oximetry in all children with ALRI [38–39]. This indication recognises the inaccuracy and unreliability of clinical signs to detect cases who need oxygen, together with the role of oxygen as an essential treatment for pneumonia [39]. Two systematic reviews highlighted that neither single nor combined symptoms and signs are sufficiently effective for predicting hypoxaemia among young children with ALRI [39–41].

Currently, there are different types of indications regarding at which Sp02 value to start oxygen therapy [38–47]. WHO recommends starting oxygen therapy when the saturation rate is below 90%, when at an altitude < 2,500 meters above sea level [38–39]. Other guidelines on acute respiratory conditions indicate as a threshold to start oxygen therapy either a value of SpO2 < 90% [42–44] or < 92% [45–46], or between 90–92% [47]. Direct evidence in support of any specific Sp02 threshold for starting supplementation with oxygen is lacking: a systematic review could not identify any study comparing outcomes of children receiving oxygen at different cut-offs of hypoxaemia [48]. Results of our review indicate that both the category of children with an SpO2 < 90% (OR 5.47, 95% CI 3.93 to 7.63) and children with SpO2 < 92% (3.66, 95% CI 1.42 to 9.47) have an increased risk of mortality, without significant difference in the ORs between the two Sp02 thresholds, and with similar sesults both at low altituides and at moderate altitudes (<2,500 meters above sea level). This suggests that even children with SpO2 below 92% may benefit from oxygen therapy.

For children living at high altitude (> 2,500 meters above sea level), WHO recommends a lower cut-off for given oxygen, such as SpO2 < 87% [38–39]. This review identifies only a small study (255 children) reporting on a population in Colombia living at an altitude > 2,500 meters, and evaluating an Sp02 of 90%, without any significant association with increased mortalty risk (OR 1.51, 95%CI 0.75 to 3.04). This may suggest that the use of Sp02 thresholds lower than 90% is appropriate, although there is no evidence in support of any precise cut-off. Further studies are therefore needed to explore the prognistic value of different cut-offs of oxygen saturation for populations living at very high altitudes (> 2,500 meters above sea level).

Effective and cheap systems for delivering oxygen have been developed and tested in low-resource countries [49–51].Policy makers should aim at improving the availability of such oxygen delivery systems together with the availability of pulse oximetry in LMIC. Alongside the provision of equipment, training and monitoring to promote effective use of available resouces is also needed. New relatively simple technologies, utilising for example smartphone devices such as pulse oxymeter, may facilitate the implementation of such practices [52].

This review adds some information to the current knowledge of prognostic factors for ALRI mortality in children in LMIC. A recent systematic review [53] highlighted that a broad range of factors are associated with increased odds of death for ALRI in children, including both child-factors (age, female sex, prematurity, low birth weight, malnutrition, inadequate breastfeeding, infections with HIV/AIDS and other co-morbidities, chronic diseases), parental factors (socio-economical status, maternal education), envoromental factors (indoor air pollution, second hand smoke exposure). The current knowledge should now be integrated in order to develop and field test comprehensive prognostic indexes–such as scoring systems including both hypoxaemia and other child, parental and environmental risk factors- in order to identify children at higher risk of ALRI mortality who should be targeted by more intensive interventions and follow up care.

### Conclusions

The results of this review support the routine evaluation of oxygen saturation rate for identifying children with ALRI at higher risk of death. Despite the lack of direct evidence in support of any specific Sp02 threshold for starting supplementation with oxygen, this review shows that both an Sp02 value of 92% and 90% equally identify children at increased risk of mortality. Further studies should focus on children living at high altitudes. Policy makers should aim at improving the availability of pulse oximetry and oxygen in LMIC.

## Supporting Information

S1 File Table A. Quality In Prognosis Studies (QUIPS) tool. Table B. Assessment of the overall risk of bias for each single study. Table C. PRISMA checklist. (DOC)
