# Supplementary material for: Hypoxaemia as a Mortality Risk Factor in Acute Lower Respiratory Infections in Children in Low and Middle-Income Countries: Systematic Review and Meta-Analysis
Source: PLoS One. 2015 Sep 15;10(9):e0136166. doi: 10.1371/journal.pone.0136166 (PMC4570717; doi:10.1371/journal.pone.0136166)
Supplement: S1 File — (DOC) [file pone.0136166.s001.doc]

**Table A. Quality In Prognosis Studies (QUIPS) tool (adapted for the study)**

| **ASSESSMENT FOR RISK OF BIAS** | | | | |
| --- | --- | --- | --- | --- |
| First author | Reviewer........................ | | | |
| **Biases** | **Issues to consider for judging overall rating of "Risk of bias"** | **Study Methods and Comments** | **Rating of reporting** | **Rating of risk of bias** |
| Assess the risk of each potential bias | These issues will guide your thinking and judgement about the overall risk of bias within each of the 6 domains. | Provide comments or excerpts to facilitate the consensus process that will follow | Y: yes  N: no  P: partial  U: unknown  NA: not applicable | HIGH MODERATE LOW  UNKNOWN |
| **1) STUDY PARTICIPATION** | The study sample adequately represents the population of interest  (i.e. The relationship between RF and death from ALRI should be the same for participants and eligible non-participants) | |  | SUMMARY |
| a. Adequate participation in the study by eligible persons (>80%) | |  |  |  |
| b. Description of the source population or population of interest | |  |  |
| c. Description of the baseline study sample | |  |  |
| d. Adequate description of the sampling frame and recruitment. | |  |  |
| e. Adequate description of the period and place of recruitment | |  |  |
| f. Adequate description of inclusion and exclusion criteria | |  |  |
| **2) STUDY ATTRITION** | The study data available (i.e. participants not lost to follow-up) adequately represent the study sample  (i.e. The relationship between RF and death from ALRI should be the same for completing and non-completing participants) | |  | SUMMARY |
| a. Adequate response rate for study participants *(> 80%)* | |  |  |  |
| b. Description of attempts to collect information on participants who dropped out | |  |  |
| c. Reasons for loss to follow-up are provided | |  |  |
| d. Adequate description of participants lost to follow-up | |  |  |
| e. There are no important differences between participants who completed the study and who did not | |  |  |
| **3) PROGNOSTIC FACTORS MEASUREMENT** | The PF is measured in a similar way for all participants  (i.e. The measurement of the RF should be the same for children who died and for those who survived) | |  | SUMMARY |
| a. A clear definition or description of the PF is provided | |  |  |  |
| b. Method of PF measurement is adequately valid and reliable (i.e. direct ascertainment; secure record, hospital record) | |  |  |
| c. Continuous variables are reported or appropriate cut points are used | |  |  |
| d. The method and setting of measurement of PF is the same for all study participants | |  |  |
| e. Adequate proportion of the study sample has complete data for the PF *(> 80%)* | |  |  |
| f. Appropriate methods of imputation are used for missing PF data | |  |  |
| **4) OUTCOME MEASUREMENT** | The outcome of interest is measured in a similar way for all participants  (i.e. The definition and ascertainment of ALRI should be the same for children who died and survivors, and for children with and without RF; for case-control studies treat the case definition as the outcome measure) | |  | SUMMARY |
| a. A clear definition of the outcome of interest is provided (including time of death) | |  |  |  |
| b. Method of outcome measurement used is adequately valid and reliable (i.e. independent blind assessment, hospital record or record linkage) | |  |  |
| c. The method and setting of outcome measurement is the same for all study participants | |  |  |
| **5) STUDY CONFOUNDING** | Important potential confounder are appropriately accounted for  (The observed effect of the RF on the death from ALRI should not be distorted by another factor related to the RF and the outcome) | |  | SUMMARY |
| a. *Most* important confounders are measured | |  |  |  |
| b. Clear definitions of the important confounders measured are provided | |  |  |
| c. Measurement of all important confounders is adequately valid and reliable | |  |  |
| d. The method and setting of confounding measurement are the same for all study participants | |  |  |
| e. Appropriate methods are used if imputation is used for missing confounder data | |  |  |
| f. Important potential confounders are accounted for in the study design (by limiting the study to specific population groups, or by matching) | |  |  |
| g. Important potential confounders are accounted for in the analysis *(by stratification, multivariate regression)* | |  |  |
| **6) STATISTICAL ANALYSIS AND PRESENTATION** | The statistical analysis is appropriate, and all primary outcomes are reported | |  | SUMMARY |
| a. Sufficient presentation of data to assess the adequacy of the analytic strategy | |  |  |  |
| b. Strategy for model building is appropriate and is based on a conceptual framework or model | |  |  |
| c. The selected statistical model is adequate for the design of the study | |  |  |
| d. There is no selective reporting of results (*based on the study protocol, if available, or on the method section* ) | |  |  |

**Table B. Assessment of the overall risk of bias for each single study**

| **Number of domains out of the total 6 domains in each category** | | | **OVERALL RISK OF BIAS** |
| --- | --- | --- | --- |
| **Low** | **Moderate/Unknown** | **High** |  |
| **6** | **0** | **0** | **LOW RISK** |
| **4 or 5** | **1 or 2** | **0** |
| **3** | **3** | **0** | **MODERATE RISK** |
|  | **1** | **1** |
|  |  | **2 or more** | **HIGH RISK** |
|  | **4 or more** |  |

**Table C. PRISMA Checklist**

| **Section/topic** | **#** | **Checklist item** | **Reported on page #** |
| --- | --- | --- | --- |
| **TITLE** | | |  |
| Title | 1 | Identify the report as a systematic review, meta-analysis, or both. | 1 |
| **ABSTRACT** | | |  |
| Structured summary | 2 | Provide a structured summary including, as applicable: background; objectives; data sources; study eligibility criteria, participants, and interventions; study appraisal and synthesis methods; results; limitations; conclusions and implications of key findings; systematic review registration number. | 2 |
| **INTRODUCTION** | | |  |
| Rationale | 3 | Describe the rationale for the review in the context of what is already known. | 3 |
| Objectives | 4 | Provide an explicit statement of questions being addressed with reference to participants, interventions, comparisons, outcomes, and study design (PICOS). | 3-4 |
| **METHODS** | | |  |
| Protocol and registration | 5 | Indicate if a review protocol exists, if and where it can be accessed (e.g., Web address), and, if available, provide registration information including registration number. | 3 |
| Eligibility criteria | 6 | Specify study characteristics (e.g., PICOS, length of follow-up) and report characteristics (e.g., years considered, language, publication status) used as criteria for eligibility, giving rationale. | 3-4 |
| Information sources | 7 | Describe all information sources (e.g., databases with dates of coverage, contact with study authors to identify additional studies) in the search and date last searched. | 3-4 |
| Search | 8 | Present full electronic search strategy for at least one database, including any limits used, such that it could be repeated. | Box 1 |
| Study selection | 9 | State the process for selecting studies (i.e., screening, eligibility, included in systematic review, and, if applicable, included in the meta-analysis). | 4  Figure 1 |
| Data collection process | 10 | Describe method of data extraction from reports (e.g., piloted forms, independently, in duplicate) and any processes for obtaining and confirming data from investigators. | 5 |
| Data items | 11 | List and define all variables for which data were sought (e.g., PICOS, funding sources) and any assumptions and simplifications made. | 5 |
| Risk of bias in individual studies | 12 | Describe methods used for assessing risk of bias of individual studies (including specification of whether this was done at the study or outcome level), and how this information is to be used in any data synthesis. | 5 |
| Summary measures | 13 | State the principal summary measures (e.g., risk ratio, difference in means). | 5 |
| Synthesis of results | 14 | Describe the methods of handling data and combining results of studies, if done, including measures of consistency (e.g., I^2^) for each meta-analysis. | 4-6 |

Page 1 of 2

| **Section/topic** | **#** | **Checklist item** | **Reported on page #** |
| --- | --- | --- | --- |
| Risk of bias across studies | 15 | Specify any assessment of risk of bias that may affect the cumulative evidence (e.g., publication bias, selective reporting within studies). | 4-5 |
| Additional analyses | 16 | Describe methods of additional analyses (e.g., sensitivity or subgroup analyses, meta-regression), if done, indicating which were pre-specified. | 5-6 |
| **RESULTS** | | |  |
| Study selection | 17 | Give numbers of studies screened, assessed for eligibility, and included in the review, with reasons for exclusions at each stage, ideally with a flow diagram. | Figure 1 |
| Study characteristics | 18 | For each study, present characteristics for which data were extracted (e.g., study size, PICOS, follow-up period) and provide the citations. | 6  Table 1 |
| Risk of bias within studies | 19 | Present data on risk of bias of each study and, if available, any outcome level assessment (see item 12). | Table 2 |
| Results of individual studies | 20 | For all outcomes considered (benefits or harms), present, for each study: (a) simple summary data for each intervention group (b) effect estimates and confidence intervals, ideally with a forest plot. | 7  Figure 2 |
| Synthesis of results | 21 | Present results of each meta-analysis done, including confidence intervals and measures of consistency. | 7  Figure 2 |
| Risk of bias across studies | 22 | Present results of any assessment of risk of bias across studies (see Item 15). | Table 2 Figure 5 |
| Additional analysis | 23 | Give results of additional analyses, if done (e.g., sensitivity or subgroup analyses, meta-regression [see Item 16]). | 7  Table 3,4  Figure 3,4 |
| **DISCUSSION** | | |  |
| Summary of evidence | 24 | Summarize the main findings including the strength of evidence for each main outcome; consider their relevance to key groups (e.g., healthcare providers, users, and policy makers). | 9-11 |
| Limitations | 25 | Discuss limitations at study and outcome level (e.g., risk of bias), and at review-level (e.g., incomplete retrieval of identified research, reporting bias). | 9-11 |
| Conclusions | 26 | Provide a general interpretation of the results in the context of other evidence, and implications for future research. | 9-11 |
| **FUNDING** | | |  |
| Funding | 27 | Describe sources of funding for the systematic review and other support (e.g., supply of data); role of funders for the systematic review. | None specific funding |

*From:*  Moher D, Liberati A, Tetzlaff J, Altman DG, The PRISMA Group (2009). Preferred Reporting Items for Systematic Reviews and Meta-Analyses: The PRISMA Statement. PLoS Med 6(6): e1000097. doi:10.1371/journal.pmed1000097

For more information, visit: **www.prisma-statement.org**

.
